# Use of the lichen *Xanthoria mandschurica* in monitoring atmospheric elemental deposition in the Taihang Mountains, Hebei, China

**DOI:** 10.1038/srep23456

**Published:** 2016-04-19

**Authors:** Hua-Jie Liu, Liang-Cheng Zhao, Shi-Bo Fang, Si-Wa Liu, Jian-Sen Hu, Lei Wang, Xiao-Di Liu, Qing-Feng Wu

**Affiliations:** 1 College of Life Sciences, Hebei University, Baoding, Hebei 071002, China; 2 Hebei Geological Laboratory, Baoding, Hebei 071051, China; 3 Chinese Academy of Meteorological Sciences, Zhongguancun South Street 46, Beijing, 100081, China

## Abstract

Air pollution is a major concern in China. Lichens are a useful biomonitor for atmospheric elemental deposition but have rarely been used in North China. The aim of this study was to investigate the atmospheric depositions of 30 trace elements (Al, Ba, Ca, Cd, Ce, Co, Cr, Cs, Cu, Fe, K, La, Mg, Mn, Mo, Na, Ni, P, Pb, Rb, Sb, Sc, Sm, Sr, Tb, Th, Ti, Tl, V and Zn) in a region of the Taihang Mountains, Hebei Province, China using lichens as biomonitors. Epilithic foliose lichen *Xanthoria mandschurica* was sampled from 21 sites and analyzed using inductively coupled plasma mass spectrometry (ICP-MS). The results show that 1) eight elements (Cd, Cr, Cu, Mo, P, Pb, Sb and Zn) are of atmospheric origin and are highly influenced by the atmospheric transportation from the North China Plain, as well as local mining activities, while 2) the remaining 22 elements are primarily of crustal origin, the concentration of which has been enhanced by local mining and quarrying activities. These results clearly validate the applicability of lichens in biomonitoring of atmospheric elemental deposition and demonstrate the spatial pattern for air pollution in the region.

The concentration of elements, particularly trace elements, in lichens has been used as an important parameter in biomonitoring of atmospheric deposition due to the direct correlation between trace element concentration in lichens and the environment^1^,^2^. Lichens obtain water and nutrients mainly from dry and wet deposition because they lack of roots, a vascular system and a protective cuticle^3^. Therefore, pollutant levels in lichen tissues originate primarily from the atmosphere^3^,^4^. Furthermore, the widespread distribution of lichens, combined with their low growth rate, long lifespan, high accumulation capability of heavy metals and high dependence of metabolism on atmospheric exchanges, make it possible to draw important information from lichens for large-scale spatial and long-term temporal patterns of atmospheric metal deposition^2^,^5^,^6^,^7^,^8^,^9^,^10^,^11^,^12^.

With rapid urbanization and industrialization in recent decades, air pollution associated with atmospheric metals has become an increasing concern in China, especially in the North China Plain where many large cities and industrial complexes are located. This concern has received national attention due to the adverse effects on human and ecosystem health^13^. Air pollution monitoring systems and instruments have been installed in recent years in China, situated mainly in urban or industrial sites. Usually, however, these systems can only detect a limited number of pollutants (mainly CO, SO_X_, NO_X_ and dust) while the data are limited in quantity so as to preclude the determination of spatial trends of atmospheric pollutants. This is particularly true in the Taihang Mountains, where air quality has been influenced by the long-range transport of atmospheric pollutants and local anthropogenic emissions, but levels and spatial patterns of atmospheric trace metal deposition have not been understood. Lichens, therefore, particularly the common and widespread species, could be an alternative or complement to instrumental methods for this purpose^2^,^3^. However, studies in this respect have not been conducted in this region to date.

The objectives of the present study are 1) to identify the origin of trace elements in lichens and 2) to map the spatial patterns of atmospheric elemental deposition measured by lichen elemental composition. Element concentrations were measured in the lichen *Xanthoria mandschurica* collected from 21 sites in Taihang Mountains (Fig. 1). This collection is a starting point to establish a regional GIS-based database of element concentrations in space and time for future large-scale and long-term, integrated elaborations.

## Results

### Elemental concentration and enrichment factor (EF)

Table 1 shows that the elemental concentrations in lichens were distributed in the following order: Fe > Ca > Al > K > Mg > Na > P > Ti > Mn > Cr >  Zn > Ba > Pb > Sr > Cu > V > Ce > Rb > Ni > La > Mo > Th > Co > Sm > Sc > Cs > Cd > Sb > Tl > Tb. The results of EFs are given in Table 1.

Al was selected to calculate EFs according to Equation 1 (see methods sections) because it is the most widely used element in lichen studies, and its concentration is constant in environmental samples within the study area, having a coefficient of variation (CV) of 14.0%, 9.8% and 8.8% for rocks, top soils and deep soils, respectively. The upper continental crust (UCC) normalized EF (EF_UCC_) is a measure of the relative contribution of crustal input. EF_UCC_ values > 5 may suggest a significant atmospheric contribution, whereas lower EFs could indicate a crustal origin^3^. The local surface rock (SR) normalized EF (EF_SR_) is a measure of the relative contribution of SR input. Both of the EFs can take into consideration the effects of secondary pollution on lichen element composition from contaminated soils.

According to EF_UCC_ values, the elements can be classified into two groups G1 (EF_UCC_ < 5) and G2 (EF_UCC_ > 5; Table 1). The G1 comprises 22 metals (Al, Ba, Ca, Ce, Co, Cs, Fe, K, La, Mg, Mn, Na, Ni, Rb, Sc, Sm, Sr, Tb, Th, Ti, Tl and V). They were not enriched relative to UCC and local SRs (EF_UCC_s and EF_SR_s < 5, Table 1), except Cs, Ni and Tl with an EF_SR_ > 5 (Table 1). For all of these elements, barring Ba, Cs, Mn, Ni, Rb, Sr and Tl, a significant, positive correlation between lichen and rock/soil samples was found and is shown in Table 1. In stark contrast, no significant correlations were found for the G2 elements (Cd, Cr, Cu, Mo, P, Pb, Sb and Zn) which were enriched relative to the UCC and local SRs (EF_UCC_s and EF_SR_s > 5, Table 1), suggesting the importance of other input sources.

### Cluster analysis (CA)

The Z-score standardized concentrations of lichen elements were subjected to CA (Euclidean distances, Ward’s criterion) to identify the correlations among them. Eight clusters were distinguished at a Euclidean distance of 6.0 (Fig. 2). Phosphorus was not significantly correlated with any other elements (p > 0.05), and thus was separated from other clusters. Elements in Clusters A, B, C and H belong to G1, whereas those in Clusters E, F and G belong to G2. Cluster D comprises four metals in G1 (Co, Mn, Ni and Th) and two metals (Cr and Mo) in G2.

### Spatial patterns of lichen elements

Elements in a cluster are often positively inter-correlated (mostly r > 0.60, p < 0.05; Pearson correlation analysis), indicating that they had similar spatial patterns. For the sake of simplicity and brevity, we mapped spatial patterns at cluster scale by using a combination of the Hasse diagram technique (HDT) and GIS techniques described by Pirintsos *et al*.^14^. The sampling sites were ranked based on the raw concentrations and EF_SR_s using HDT in order to present partial order relations^14^. The outputs of HDT were subjected to Kriging interpolation and mapped to visualize spatial patterns.

The concentration patterns for the G1 elements are given in Fig. 3a–f. Ca, Mg, Cs and Tl show a decreasing pattern from north to south (Fig. 3a) while a reverse pattern is observed for K, Na, Sm and Tb (Fig. 3b). Concentrations of Ce, La and Rb were high near Fuping (sites S8, 9, 10, 14) and Tangxian (S11; Fig. 3c). The patterns for clusters A and D were similar, characterized by higher concentrations near Fuping (S9), Tangxian (S11) and Jingxing (S21), and lower concentrations at sites S1–7 (Fig. 3d,e).

The concentration and EF_SR_ patterns for the G2 elements are given in Fig. 4a–h. The concentration pattern for Cr and Mo (Fig. 4a) was similar to the EF_SR_ pattern (Fig. 4b), which was also highly similar to the concentration pattern for Mn, Ni, Co and Th (Fig. 3e), as expected from their close relationships (Fig. 2). Concentrations for Cu and Sb were highest near Tangxian (S11 and S12) and also high along the line from S17 to S4 (Fig. 4c). Concentrations for Cd, Pb and Zn show a different pattern with a decreasing trend from east to west and a northwestward tailing from S19 (Fig. 4e). This concentration pattern was also observed for P, which however, showed a high concentration at sites S8 and S10 (Fig. 4g). EF_SR_ patterns for Cd, Cu, P, Pb, Sb and Zn were more or less similar, with a westward decreasing trend from S1, S2, S5 and S6, and a northwestward tailing from S19 (Fig. 4d,f,h).

## Discussion

### Origin of lichen elements: crustal vs. atmospheric

All G1 metals were derived mainly from crustal materials, as indicated by the EF_UCC_s and EF_SR_s of <5 (Table 1), whereas G2 elements were possibly of atmospheric origin (EF_UCC_s and EF_SR_s >5; Table 1). Because element concentration in lichens is often correlated with environmental levels^1^,^2^, significant, positive correlations of G1 elements between lichens and environmental samples could also suggest a considerable crustal input, whereas the lacking of such correlations of G2 elements may imply an atmospheric origin (Table 1). Because a good correlation between lichen elemental concentrations may suggest a common origin^7^, this inference is also supported by the CA results. Most G1 elements are in the clusters A, B, C and H, while most G2 elements belong to other clusters (Fig. 2).

Our conclusion is in strong agreement with previous studies where lichens were used as biomonitors of atmospheric deposition. Most of the G1 elements in lichens have been regarded as crustal in origin, while Cd, Cr, Mo, Pb, Zn in G2 as atmospheric in origin^12^,^15^,^16^,^17^,^18^,^19^,^20^,^21^,^22^,^23^.

### Concentrations, sources and spatial patterns for G1 elements

The mean concentrations of G1 elements in lichens in this study were generally higher than or at the upper range of values found in the literature for different lichen species from different locations, even in urban and industrial sites^5^,^7^,^8^,^9^,^12^,^15^,^19^,^20^,^22^,^24^,^25^. This finding could suggest a much higher crustal particulate input in the study area due to a greater impact of human activities on natural element cycles. A variety of human activities such as mining, quarrying and processing of stones in the study area has released a substantial quantity of mineral particulates into the atmosphere. Levels of Ca, Cs, Mg and Tl in lichens have been highly influenced by stone quarrying and processing. Concentration patterns for these metals are different from other crustal elements, with high concentrations at sites S1 to S9 and S5 to S12 (Fig. 3a), where many stone quarrying and processing factories are located. Because stone materials are mostly mined from surface quarries, a significant, positive correlation between lichens and SRs for Ca and Mg is reasonable (Table 1).

Concentration patterns for K, Na, Sm and Tb (Fig. 3b) are mainly a reflection of their composition in soils, as indicated by the significant correlations between lichens and soils (Table 1). This finding may be observed for Ce and La, both of which are also positively correlated between lichens and soils but not between lichens and SRs (Table 1). However, considering the higher concentrations found in this study in contrast to the literature, the influence of northward transportation of windblown soil particulates from Shijiazhuang-Jingxing and Tangxian could be considerable for K, Na, Sm and Tb. The effects of mining activities near Fuping and Tangxian may be responsible for the high concentrations of Ce and La in lichens (Fig. 3c).

The other 11 crustal elements (Al, Ba, Co, Fe, Mn, Ni, Sc, Sr, Th, Ti and V) show roughly similar patterns, characterized by higher concentrations near regions with intensive mineral mining activities, for instance, at Fuping (sites S9 to S17), Pingshan (S21) and Tangxian (S11; Fig. 3d,e). Correlations were insignificant between lichens and SRs for these metals (except Sc) and were significant for only five metals (Al, Co, Fe, Th and Ti) between lichens and shallow soils (Table 1). This could be largely attributable to the strong influence of element input from deep mineral layers.

### Concentrations, sources and spatial patterns for G2 elements

The mean concentrations for G2 elements in lichens are also higher than or at the upper range of most values found in the literature for different lichen species from diverse ecosystems^5^,^6^,^7^,^8^,^9^,^10^,^11^,^12^,^15^,^16^,^17^,^18^,^19^,^20^,^21^,^22^,^23^,^24^,^25^. The main sources for all of these elements in lichens are a combination of atmospheric transportation from the North China Plain and local mining activities in mountainous regions.

The influence of local mining activities are evident from Fuping to Jingxing (Site S9 to S20), where seven metals (Cd, Cr, Cu, Mo, Pb, Sb and Zn) are frequently mineralized in deep rock layers to varying extents^26^, and uncontrolled mining activities have been operated by many small, private mines in recent decades. The patterns for Cr and Mo are characterized by a higher concentration and EF_SR_ at sites S9, S10, S17 and S20 (Fig. 4a,b). These patterns are very similar to that of Co, Mn, Ni and Th (Fig. 3e) and, to a lesser extent, for the crustal elements Al, Ba, Fe, Sc, Sr, Ti and V (Fig. 3d). This finding suggests a strong influence of mineral particulate input released from deep mineral layers. This influence was also observed in Cu and Sb, especially at sites along the line from S17 to S4, where similar concentrations but lower EF_SR_s were evident when compared with sites near the North China Plain (Fig. 4c,d). At sites S9, S20 and S21, the higher concentrations but lower EF_SR_s for Cd, Pb and Zn also suggest this influence (Fig. 4e,f).

According to the EF_SR_ patterns (Fig. 4b,d,f,h), atmospheric transportation from the North China Plain with the prevailing southeast and east winds could also be an important source for Cd, Cu, P, Pb, Sb and Zn, especially at sites located near the North China Plain. The EF_SR_s for these elements show a westward decrease from Baoding and a northwestward decrease from Shijiazhuang, suggesting a significant contribution from transportation of atmospheric pollutants from both cities. For Cr and Mo, a northward decreasing trend of EF_SR_ from sites S5, S11 and S12 (Fig. 4b) reflects atmospheric transportation from Shijiazhuang.

A strong influence of atmospheric transportation from the North China Plain on the heavy metal patterns in the lichen is reasonable because heavy metals are common atmospheric pollutants at urban and industrial sites located on the North China Plain. Hebei Province is notorious for having the highest crude steel production in China in recent years, accounting for approximately 25% of the total national output. Hebei is also among the several provinces that release the largest amounts of Cr, Cu, Pb and Zn, accounting for approximately 7.1%, 6.7%, 9.3% and 11.3% of the national totals, respectively^13^, which has been attributed to intense traffic along with heavy industrial and domestic emissions^13^,^27^,^28^,^29^. The intensive agricultural operations, especially the use of pesticides and fertilizers, could be another important source for heavy metals and P in the air and soil. Finally, the resuspension and transportation of polluted soil from farming, urban and industrial areas may also be important contributors for the enriched elements in lichens.

It should be noted that although influenced by atmospheric transportation from the North China Plain, P is not associated with any other elements in the CA (Fig. 2). We feel this does not necessarily imply a different source input, but rather an interaction between atmospheric transportation from the North China Plain and biological regulation in lichens, as P is a bio-essential nutrient that can be absorbed from the soil and biologically regulated by plants.

Our findings demonstrate that a combination of atmospheric transportation from the North China Plain and local industrial operations led to substantial inputs of chemical elements into ecosystems, potentially posing detrimental effects on human and ecosystem health. Therefore, intensive monitoring and strict controls of relevant industrial activities should be implemented in this most seriously metal-polluted area in China, for which a clear understanding of the source-sink relationship and spatiotemporal pattern of important chemical elements would be a prerequisite.

## Methods

### Study area

The study area (38°14′-39°15′N, 113°38′-115°17′E) lies in the Taihang Mountains in Hebei Province. The area covers approximately 8,000 km^2^ with an increasing elevation from east to west (approximately 100 m to 2,000 m, respectively). The climate is continental temperate monsoon, with a mean annual rainfall of approximately 550 mm. The prevailing winds are from the southeast in summer and northwest in winter. In addition to intensive agricultural cultivation and a well-developed road network supporting heavy traffic, the area is also characterized by intense industrial operations including mining, quarrying, road construction and cement production, etc. In China, this has led to severe air pollution in the form of aerosol smog, particulate storms and acid rain.

### Sampling strategies

**Figure 1:**
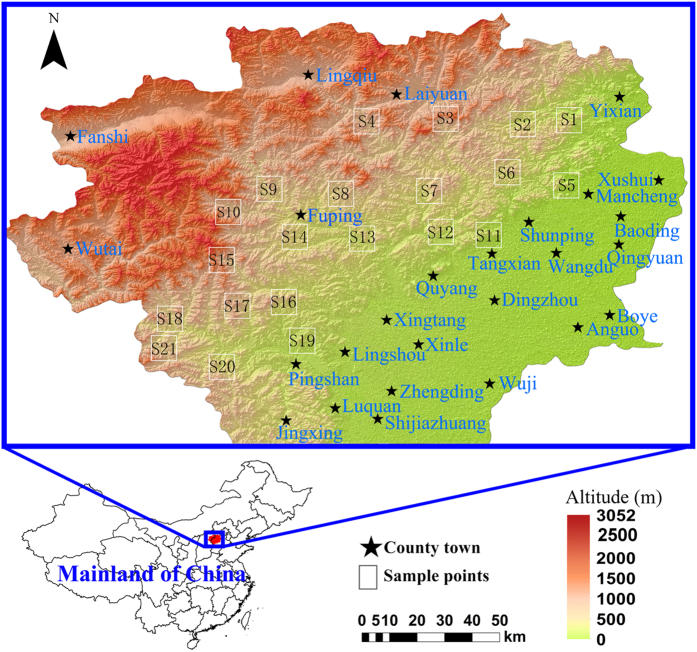
Location of the sampling sites. The white hollow squares denote the sampling sites while the black stars represent cities. The map was prepared using ArcGIS 10.2 software (ESRI Inc, Redlands, CA, USA) with 3D visualization of the DEM data to illustrate the geomorphic features. For details of the DEM data, see the Methods section.

**Figure 2:**
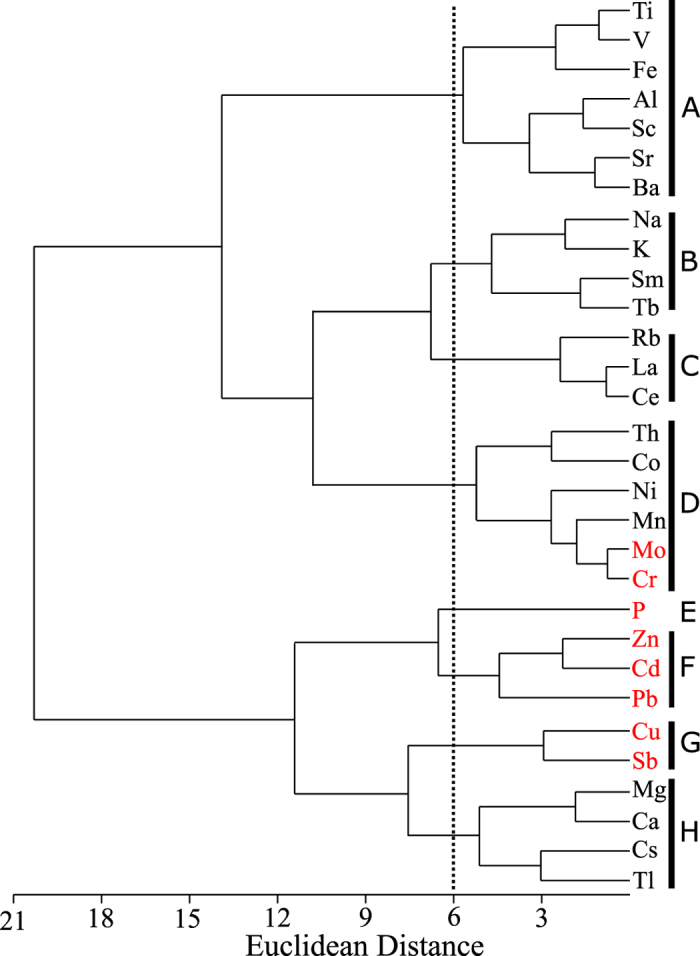
Dendrogram obtained using Euclidean distance measure and Ward’s method on the basis of the Z-scored lichen element concentrations from 21 sites. Elements in black and red belong to group G1 and G2, respectively. The broken line signifies clusters formed at a Euclidean distance equal to 6.0.

Twenty-one sampling sites were selected at an interval of 15–20 km (Fig. 1) according to the following protocols: 1) mountainous areas rather than flat lands; 2) grassland ecosystem; 3) a relative height of 100 to 200 m to the foot of the local hills or mountains (elevations from 200 to 1000 m) and 4) at least 2 km from major roads, industries and settlements in addition to being at least 0.5 km from county roads and farms. The purpose of these protocols was to minimize factors that could potentially influence lichen elemental composition, such as local anthropogenic emissions, climate, landscape and vegetation. *Xanthoria mandschurica* was collected at each sampling site over the course of two weeks in October 2013. At each site, in an area of 1 km^2^, at least 50 individuals of *X*. *mandschurica* with diameters of 3–5 cm were randomly collected on rocks at a height of 0.5 m above ground level and 5 m from trees or shrubs (if present). SRs were randomly extracted using a hammer, while shallow soil (0–5 cm) and deep soil (20–50 cm) were extracted using a soil gauge. The composite samples were mixed to represent the average status of elemental composition in the site, and then preserved in a paper bag.

### Sample preparation and chemical analysis

The outermost part of the lichen thalli (1–1.5 cm) was detached and carefully cleaned for analysis. Because the outermost, 2–5 mm part of the lichen corresponds to parts of the thallus that are one year in age^21^,^30^, this allows a comparison of recently accumulated elements by minimizing the effects of age^31^. Lichen samples were air-dried at room temperature in paper bags and then oven-dried at 70 °C for 72 h to a constant weight. Soil samples were also thoroughly cleaned and then dried using the above method. All samples were ground and homogenized in a grinding mill equipped with Tungsten Carbide jars (Retsch MM400; Retsch GmbH, Haan, Germany).

200–300 mg of each sample were mineralized by microwaves in high pressure Teflon vessels in a mixture of HNO_3_ and H_2_O_2_ for lichens, and in a mixture of HNO_3_, HF, HCl, and HClO_4_ for both rock and soil samples. Thirty elements (Al, Ba, Ca, Cd, Ce, Co, Cr, Cs, Cu, Fe, K, La, Mg, Mn, Mo, Na, Ni, P, Pb, Rb, Sb, Sc, Sm, Sr, Tb, Th, Ti, Tl, V and Zn) were analyzed at Hebei Geological Laboratory by inductively coupled plasma mass spectrometry (ICP-MS; Agilent 7700X; Agilent Technologies, Tokyo, Japan). The analytical quality of the results was evaluated against the following reference materials: the national reference materials of GBW07359, GBW07361, GBW07362 and GBW07365 for rocks; GBW07451, GBW07452 and GBW07457 for soils; and GBW10014 (cabbage), GBW10015 (spinach), GBW10052 (green tea; all materials mentioned above were issued by Institute of Geophysical and Geochemical Exploration, Chinese Academy of Geological Sciences) and IAEA-336 (lichen, issued by the International Atomic Energy Agency) for lichens. The measured element concentrations were within the confidence intervals of the certified/suggested value. Analytical precision was generally <5% as judged from frequent, duplicate analyses.

### Calculation of EF

EF has been widely used in determining elemental sources in lichens^3^. In this study, EF was calculated by normalizing elemental concentrations in lichen to the averaged values of local SRs or the means of UCC, described as:
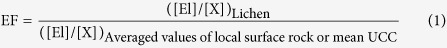
where “X” is the concentration of the chosen reference element and “El” is the element under consideration. The square brackets denote concentration. The subscripts denote the sample type. For the natural abundance of elements for the UCC standard, see Rudnick and Gao^32^.

### Statistical analysis

A one-sample Kolmogorov-Smirnov test was conducted to test the normal distribution for each element. When the data were normally distributed, a Pearson correlation analysis was conducted between the elements of lichens and local SRs, top soils and deep soils; otherwise, a Spearman rank-order correlation analysis was performed. A CA of the lichen elemental concentration was performed using the software Past 3.10 (Ø. Hammer, Nov. 2015. http://folk.uio.no/ohammer/past/) using the Euclidean distance of the Z-score standardized variables to measure dissimilarities and Ward’s criterion to construct the hierarchical tree.

**Figure 3:**
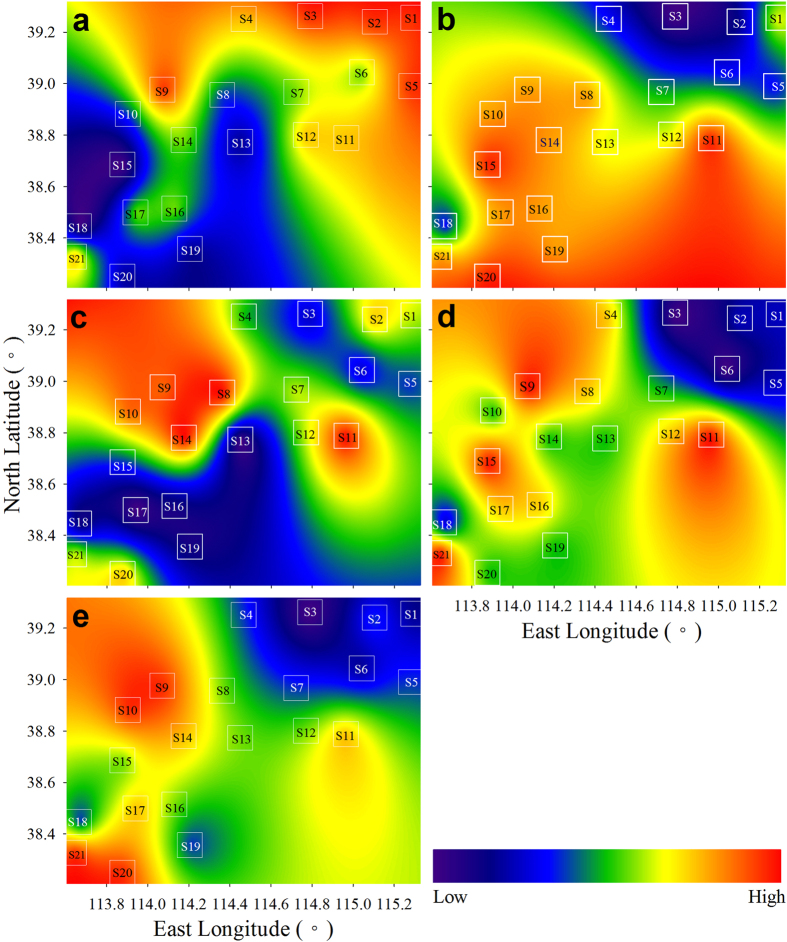
Spatial patterns for lichen element concentrations based on the Kriging interpolation of the HDT outputs of the sampling sites. (**a**) Ca, Cs, Mg and Tl (cluster H). (**b**) Na, K, Sm and Tb (cluster B). (**c**) Ce, La and Rb (cluster C). (**d**) Al, Ba, Fe, Sc, Sr, Ti and V (cluster A). (**e**) Co, Mn, Ni and Th (cluster D). The white squares denote sampling sites. The lichen is *Xanthoria mandschurica*. The HDT outputs, produced using the software Dart 2.0.5 (Talete srl 2007), were subjected to Kriging interpolation using Past 3.10 (Ø. Hammer, Nov. 2015). Maps of the Kriging results were drawn using SigmaPlot 12.5 (Systat Software, Inc., San Jose, CA, USA). For mapping details see Fig. 1.

**Figure 4:**
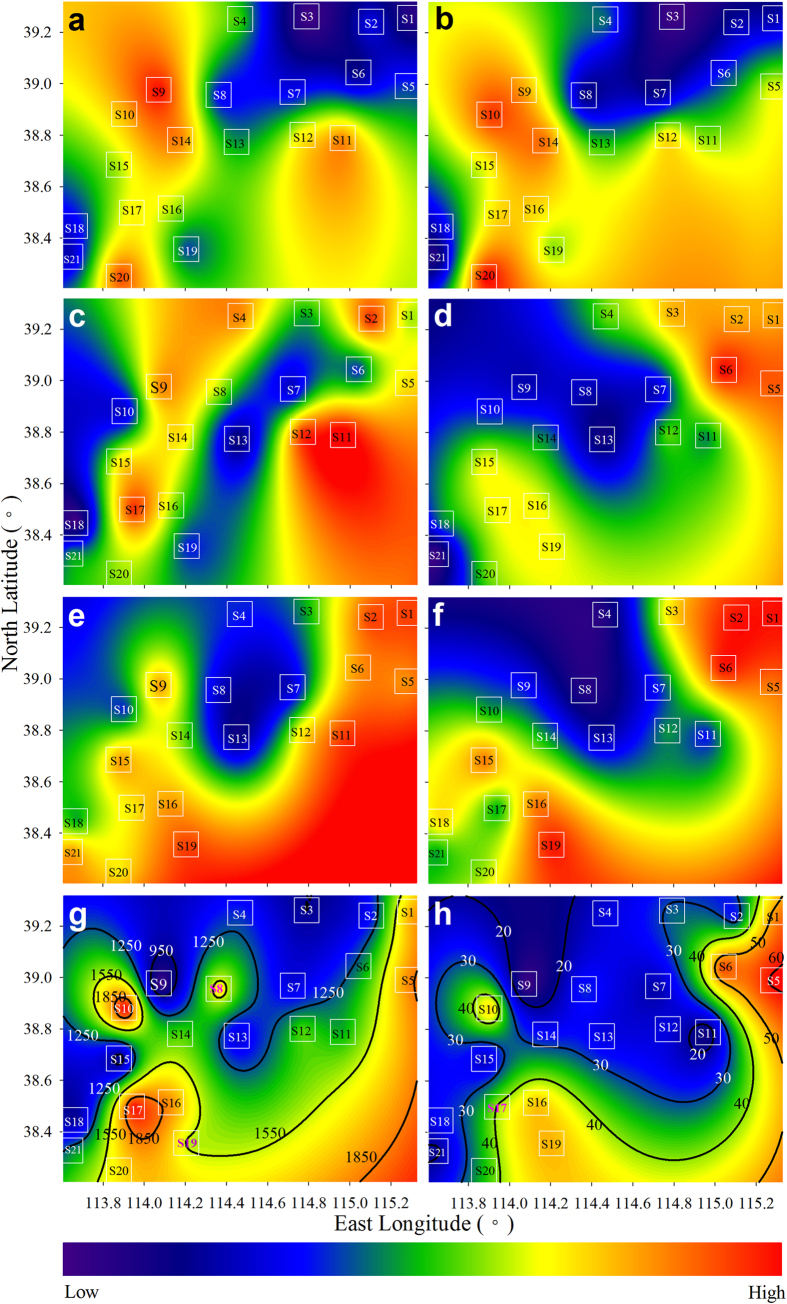
Spatial patterns of concentration and EF for lichen elements. (**a–f**) based on the Kriging interpolation of the HDT outputs of sampling sites. (**g–h**) based on the Kriging interpolation of the raw data. (**a**) Cr and Mo (concentration). (**b**) Cr and Mo (EF_SR_). (**c**) Cu and Sb (concentration). (**d**) Cu and Sb (EF_SR_). (**e**) Cd, Pb and Zn (concentration). (**f**) Cd, Pb and Zn (EF_SR_). (**g**) P (concentration in ug·g^−1^). (**h**) P (EF_SR_). EF_SR_, EF normalized to averaged local SRs. The white squares denote sampling sites. The lichen is *Xanthoria mandschurica*. The HDT outputs, produced using the software Dart 2.0.5 (Talete srl 2007), were subjected to Kriging interpolation using Past 3.10 (Ø. Hammer, Nov. 2015). Maps of the Kriging results were drawn using SigmaPlot 12.5 (Systat Software, Inc., San Jose, CA, USA). For mapping details see Fig. 1.

The map in Fig. 1 was prepared using ArcGIS 10.2 software (ESRI Inc, Redlands, CA, USA; http://www.esri.com) for 3D visualization of digital elevation map (DEM) data that were derived from the ASTER Global Digital Elevation Model (ASTER GDEM) with a horizontal accuracy of 30 m and obtained from Geospatial Data Cloud (http://www.gscloud.cn/). Other maps (Figs 3 and 4) were produced using a combination of HDT and GIS techniques to elucidate spatial patterns as described by Pirintsos *et al*.^14^. HDT was conducted separately on the seven clusters of lichen elements, in terms of raw concentration and EF_SR_, to rank the sampling sites, using the software Dart 2.0.5 (Talete srl 2007. http://www.talete.mi.it/products/dart_description.htm) according to the manual^33^. The HDT outputs were subjected to Kriging analysis in Past 3.10, and maps of the Kriging results were drawn using SigmaPlot 12.5 (Systat Software, Inc., San Jose, CA, USA). The Kriging analysis was conducted according to the manual^34^ using a spherical model with the “nugget” = 0 and “range” = 1.5 to ensure accurate estimates; in this process, the “bins” was set to 5 and the “scale” was manually adjusted in order to obtain the smallest possible SSerror.

## Additional Information

**How to cite this article**: Liu, H.-J. *et al*. Use of the lichen *Xanthoria mandschurica* in monitoring atmospheric elemental deposition in the Taihang Mountains, Hebei, China. *Sci. Rep.* **6**, 23456; doi: 10.1038/srep23456 (2016).

## Figures and Tables

**Table 1:** Concentrations and EFs of lichen elements and their correlations to those in soils and rocks.

		**Concentration (ug·g**^−1^)		**EF**			**Correlation**		
		**Mean**	**CV (%)**	**EF**_**SR**_	**EF**_**UCC**_	**CV (%)**	**r**_**SR**_	**r**_**TS**_	**r**_**DS**_
**Element**				**Mean**	**Mean**				
Group G1	Al	8828.31	33.85	1.00	1.00	0.00	0.22	0.44*	0.08
	Ba	90.73	43.71	0.75	1.34	26.67	−0.42	0.07	−0.06
	Ca	8906.25	49.27	4.49	3.43	60.13	0.47*	−0.22	0.43*
	Ce	17.75	60.56	2.67	2.57	51.31	0.24	0.67*	0.63*
	Co	2.81	40.57	3.45	1.56	33.91	−0.18!	0.59*	0.47*
	Cs	1.28	23.44	7.49	2.61	33.07	0.15	0.22	0.26
	Fe	9682.48	37.06	3.79	2.34	33.25	0.06	0.46*	0.37
	K	6343.17	23.85	1.81	2.70	30.94	0.67*	0.61*	0.37
	La	8.87	62.34	3.08	2.60	51.95	0.07	0.57*	0.64*
	Mg	2474.93	87.71	2.78	1.67	103.24	0.66*	0.05	−0.09
	Mn	261.63	47.35	4.45	3.08	34.38	0.22	0.36	0.26
	Na	1962.23	58.24	0.70	0.72	38.57	0.34	0.74*	0.46*
	Ni	13.37	38.89	5.57	2.72	31.60	−0.32	0.25	−0.03
	Rb	17.58	31.51	1.21	2.05	35.54	−0.01	0.10	−0.11
	Sc	1.38	35.51	1.88	0.91	18.09	0.45*	0.33	0.53*
	Sm	1.47	59.86	2.85	2.87	55.79	−0.14!	0.82*	0.72*
	Sr	33.33	45.84	0.93	0.96	22.58	−0.39	0.04	0.23
	Tb	0.21	61.90	2.92	2.87	65.41	0.00!	0.75*	0.64*
	Th	3.01	44.52	3.21	2.70	38.63	−0.14	0.72*	0.43*
	Ti	516.73	39.51	2.47	1.26	29.55	0.04	0.53*	0.34
	Tl	0.38	26.32	5.70	4.25	41.75	−0.40	−0.23	−0.33
	V	18.49	34.34	3.71	1.81	23.72	0.39	0.36	0.55*
Group G2	Cd	1.05	40.00	108.11	123.99	63.06	0.08!	0.08	0.12
	Cr	202.16	68.09	14.61	19.6	55.03	−0.08	0.22	0.14
	Cu	21.78	31.22	10.67	7.52	27.18	−0.15	−0.36	−0.27
	Mo	5.12	67.19	16.07	42.39	56.00	−0.25	0.38	0.03
	P	1379.13	28.73	32.99	21.68	43.92	−0.26	−0.24	−0.14
	Pb	48.14	47.36	25.02	29.94	71.10	−0.07	−0.08	−0.18!
	Sb	0.79	20.25	79.85	19.79	33.93	0.27	0.05	0.25
	Zn	102.12	36.40	19.10	15.47	47.38	0.14!	−0.16	0.01

CV refers to coefficient of variance. EF_SR_ refers to EF normalized to averaged local SRs. EF_UCC_ refers to EF normalized to the mean UCC. The r_SR_, r_TS_ and r_DS_ represent the correlation of the lichen with local SRs, top soils and deep soils, respectively. In the correlation rows, *denotes a significant correlation in elemental concentration between lichen and the environmental samples at p ≤ 0.05; ‘!’ denotes the significance was tested by a Spearman rank-order correlation analysis; otherwise, it was tested by a Pearson correlation test. The lichen is *Xanthoria mandschurica*. n = 21.
